# Expanding Role of Contrast-Enhanced Ultrasound and Elastography in the Evaluation of Abdominal Pathologies in Children

**DOI:** 10.3390/diagnostics15131680

**Published:** 2025-07-01

**Authors:** Natae Fekadu Lemessa, Laith R. Sultan, Santiago Martinez-Correa, Laura May Davis, Misun Hwang

**Affiliations:** 1 Department of Radiology, Children’s Hospital of Philadelphia, Philadelphia, PA 19104, USA; lemessan@chop.edu (N.F.L.); martinezcs@chop.edu (S.M.-C.); davisl19@chop.edu (L.M.D.); 2 Department of Radiology, Perelman School of Medicine, University of Pennsylvania, Philadelphia, PA 19104, USA

**Keywords:** contrast-enhanced ultrasound, elastography, multiparametric ultrasound, pediatrics, abdominal imaging

## Abstract

Contrast-enhanced ultrasound and elastography are two ultrasound technologies that are becoming increasingly popular in the evaluation of different abdominal pathologies in children. The use of these technologies has expanded the diagnostic scope of ultrasound into areas that were traditionally covered by advanced imaging modalities such as computed tomography, magnetic resonance imaging, and fluoroscopy. In this review, we summarize the use of contrast-enhanced ultrasound and elastography in the evaluation of hepatic, renal, pancreatic, splenic, urinary tract, and scrotal pathologies in children. We describe the technical aspects, applications, and limitations, intending to make readers more acquainted with the technologies.

## 1. Introduction

Ultrasound (US) remains the first-line imaging modality for assessing most abdominal abnormalities in the pediatric population. Children often go through more advanced and expensive imaging modalities such as computed tomography (CT) and magnetic resonance imaging (MRI) when information acquired from abdominal US imaging is deemed insufficient. Unlike CT and MRI, US has an excellent safety profile devoid of complications that can arise from using potentially toxic contrast agents or the need for sedation [1,2]. Considering the vital role that US plays in the evaluation of children, the continuous innovation of novel technologies or the refinement of existing ones has a tremendous impact on the clinical care of the pediatric population. Contrast-enhanced US (CEUS) and US elastography (UE) are two developments that have expanded the scope of US diagnostics in the adult population. Although both have been utilized to varying degrees in children, there is still a paucity of literature regarding their use in this population. This paper will review the clinical applications of the two modalities in abdominal imaging in children.

## 2. Contrast-Enhanced Ultrasound (CEUS)

CEUS is a rapidly expanding US technique in children, gaining momentum after the innovation of robust second-generation US contrast agents (UCA) that enable a long evaluation time. UCA are comprised of microbubbles that resonate when insonated by US pulsation, with a resultant harmonic signal used to produce high-contrast images [3]. Because of their size (1–10 microns—similar to that of red blood cells), the bubbles remain within the circulatory system, allowing both qualitative and quantitative evaluation of the microvasculature of organs and their perfusion [4].

Currently, the only Food and Drug Administration (FDA)-approved contrast agent for use in children in the United States is Lumason/SonoVue (Bracco Diagnostics Inc., Monroe Township, NJ, USA). It is a microbubble composed of a sulfur hexafluoride inert gas encapsulated in a lipid-type A microsphere. The recommended dose of Lumason is 0.03 mL/kg (up to a maximum dose of 2.4 mL per scan) at a slow rate of 1–2 mL/s via an intravenous catheter. After administration of the contrast agent, a saline bolus is given via a three-way stopcock [5,6].

The benefits of CEUS in children are numerous. Several studies have testified to an excellent safety record of UCA in children, even better than that seen in the adult population [2,7,8,9,10]. In addition, the procedure can be done at the bedside, avoiding the risk of thermal instability in infants and the need to mobilize critically ill children. Another important advantage is that UCA are excreted via the biliary system and the lungs, with no contraindication in the setting of renal or hepatic impairment [5].

Although a multitude of uses for CEUS are reported in the literature, FDA approval so far is limited to three clinical applications [11]. These include the evaluation of focal liver lesions, cardiac chamber, and vesicoureteral reflux (VUR) [6,12]. These applications and other uses will be discussed below. The use of CEUS for many other pediatric applications beyond FDA-approved indications is considered off-label in the United States. The off-label usage of contrast is routinely performed as part of clinically indicated care in children. The incidence of serious complications, such as anaphylaxis, is extremely low, reported at less than 0.01% in pediatric populations [2,7,9]. Most adverse reactions are mild (e.g., transient headache, nausea), and there have been no known cases of renal or hepatic toxicity, as microbubbles are not nephrotoxic and are excreted via the lungs and biliary system. Consequently, CEUS is often considered safer than iodinated or gadolinium-based contrast agents in pediatric patients, especially those with renal impairment.

## 3. Clinical Applications of CEUS

### 3.1. Liver

CEUS is an invaluable tool in pediatric liver imaging, particularly in distinguishing between benign and malignant focal liver lesions—an often complex but essential differentiation for determining appropriate treatment strategies [13]. Benign lesions are typically characterized by variable wash-in patterns (i.e., hyper-, hypo-, or iso-) and absence of early wash-out [14]. In contrast, malignant liver tumors, such as hepatoblastoma and hepatocellular carcinoma, generally exhibit rapid enhancement followed by washout, though the timing of this washout can vary, with some tumors displaying delayed washout patterns [14,15]. Understanding these enhancement patterns allows for accurate diagnosis using CEUS alone, such as in the case of hemangiomas, as well as effective triage for MRI and/or guidance for intervention.

**Figure 1:**
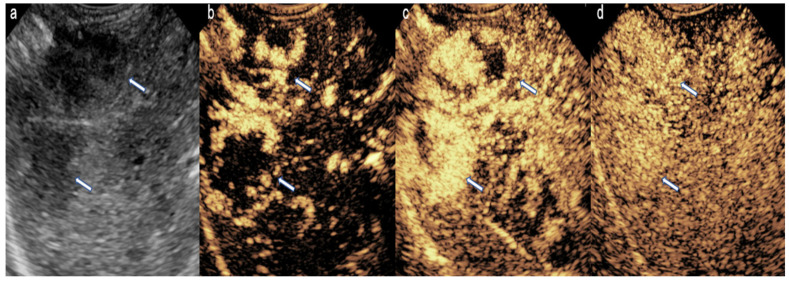
CEUS in a 24-day-old girl with hemangioma. (**a**) A transverse scan of the right lobe of the liver shows multiple hypoechoic masses (arrow). (**b**) Arterial phase shows nodular peripheral hyperenhancement. (**c**) Porto-venous phase shows hyperenhancement with progressive centripetal filling. (**d**) Delayed phase shows the absence of rapid washout.

CEUS is commonly used in the evaluation of benign liver lesions in pediatric patients, such as hemangiomas and focal nodular hyperplasia [5]. Hemangiomas, which are the most frequently occurring benign liver tumors in children, display a characteristic enhancement pattern. They show peripheral, discontinuous nodular enhancement that gradually fills in toward the center (centripetal fill-in), without washout in later phases (Figure 1). This enhancement pattern is a hallmark feature that aids clinicians in confidently identifying hemangiomas, avoiding unnecessary biopsies or interventions.

**Figure 2:**
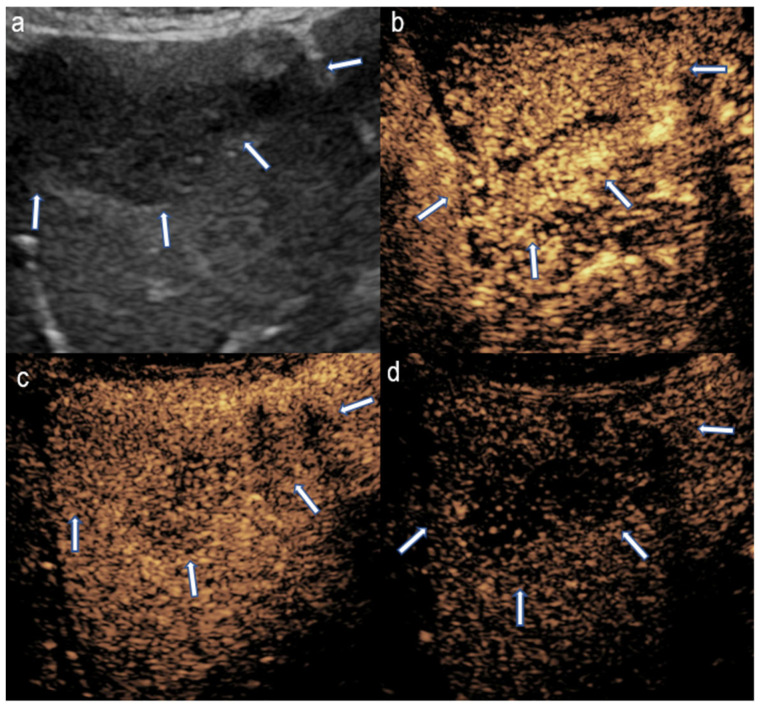
CEUS in a 16-year-old girl with hepatoblastoma. (**a**) A longitudinal scan of the liver shows a well-defined, homogenously hypoechoic mass in grayscale (arrow). (**b**) Iso- to slightly hyperintense enhancement on early arterial phase. (**c**) Hypoenhancement in porto-venous phase. (**d**) Early washout on delayed phase indicative of the malignant nature of the mass.

On the other hand, malignant liver tumors, such as hepatoblastoma—the most common pediatric liver malignancy—tend to present with a more heterogeneous enhancement pattern. Hepatoblastomas typically show rapid and heterogeneous enhancement soon after contrast injection, followed by an early washout phase, which may be an indicator of the tumor’s aggressiveness (Figure 2) [16]. While these enhancement patterns can overlap between benign and malignant lesions, the timing and characteristics of washout are often pivotal in guiding further diagnostic steps, including the need for biopsy or surgical intervention.

Beyond lesion characterization, CEUS has proved highly effective in evaluating the vascular architecture of liver lesions, an essential aspect that significantly influences both diagnosis and treatment planning [13]. In malignant tumors such as hepatoblastoma, CEUS can reveal extensive neovascularization, an indicator of the tumor’s potential for growth and metastasis. This is particularly useful for surgical planning, as the degree of vascular involvement can influence the surgical approach. Additionally, CEUS can be used to assess vascular anomalies such as hepatic hemangiomas and arteriovenous malformations. In these cases, CEUS provides real-time visualization of blood flow patterns, allowing for precise differentiation between high-flow lesions, such as arteriovenous malformations, and low-flow lesions, like hemangiomas [17]. This real-time capability provides clinicians with crucial information for treatment planning, particularly in cases where vascular lesions are associated with increased bleeding risk or other complications.

In clinical practice, the utility of CEUS is further supported by recommendations from expert panels. The American College of Radiology Pediatric LI-RADS Working Group endorses the use of CEUS for the evaluation of newly identified focal liver lesions in children, especially when conventional imaging modalities fail to provide definitive information [15]. The real-time nature of CEUS allows for dynamic assessment of contrast enhancement patterns, which can be critical in distinguishing between different types of liver lesions.

Although CEUS has established itself as a valuable tool in pediatric liver imaging, certain challenges remain. Interpreting enhancement patterns in complex liver lesions or in cases involving multiple lesions can be diagnostically challenging, requiring careful correlation with clinical findings and, at times, additional imaging modalities [16,18]. Furthermore, ensuring reproducibility and standardization in CEUS protocols across diverse clinical settings remains an area for continued research and development [19].

Despite these challenges, CEUS continues to expand its role in pediatric radiology, with ongoing advancements emphasizing its potential in non-invasive, real-time evaluation of liver lesions. Future research aimed at addressing current limitations, improving standardization, and exploring novel applications is likely to further solidify its place in clinical practice [20].

### 3.2. Bowel

CEUS has been used off-label to evaluate various pathologies of the bowel, such as inflammatory bowel disease (IBD) (i.e., Crohn’s disease and ulcerative colitis) [21,22,23], necrotizing enterocolitis (NEC) [24,25,26], and intussusception [24].

IBD developed during childhood or adolescence is responsible for 20–25% of the global disease burden [27]. The clinical course of IBD is associated with multiple complications requiring frequent follow-ups. Currently, ileocolonoscopy, upper endoscopy, MR enterography, and sometimes capsular endoscopy are used for monitoring IBD [21,28]. However, these modalities are expensive, time-consuming, and/or invasive.

Conventional US has been introduced as a complementary tool to MR enterography in evaluating IBD. During inflammation, the affected segment of the bowel thickens and becomes hyperemic, and both features are often readily detected by US and conventional Doppler US [29]. Hyperemia is a testament to the neovascularization and increased blood flow associated with IBD [24]. Most of the newly formed blood vessels, however, are too small to be detected by Doppler US. CEUS, on the other hand, has a superior ability to depict these small vessels. Although still exploratory, CEUS shows potential for distinguishing acute from chronic inflammation, monitoring treatment response, and assessing the level of inflammatory activity in newly diagnosed IBD cases [4].

**Figure 3:**
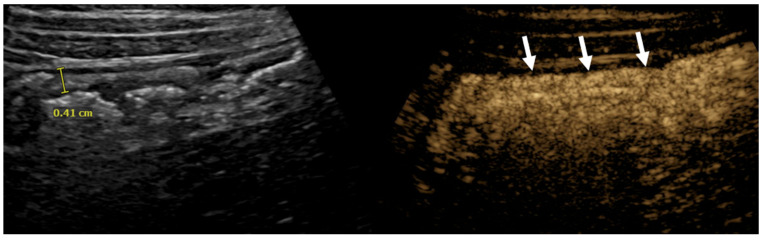
Ileocolonic Crohn’s disease in a 13-year-old boy. Dual-screen mode with simultaneous display of grayscale (**left**) and contrast-enhanced (**right**) proximal ascending colon in the longitudinal plane shows smoothly thickened proximal ascending colon with rapid wash-in and avid enhancement (arrows). The enhancement persisted in porto-venous and delayed phases.

The normal bowel follows three enhancement phases after administering UCA. Initially, there is a brief period of no enhancement that lasts 20 s, immediately followed by maximum mural enhancement. Lastly, 45–60 s after contrast administration, gradual contrast washout occurs [24]. CEUS in individuals with active IBD has shown fast and intense mural enhancement with delayed onset of contrast washout (Figure 3) [24]. This characteristic of active IBD on CEUS is used to differentiate between flares and chronic inflammation [24].

Quantitative analysis of CEUS has emerged as a pivotal tool in the assessment of Crohn’s disease, offering deeper insights into disease activity and treatment response. By meticulously analyzing parameters such as perfusion patterns and wash-in and wash-out times, CEUS provides clinicians with objective metrics to differentiate mild from moderate and moderate from severe IBD inflammation. This method enhances diagnostic accuracy and aids in tailoring therapeutic strategies, potentially improving patient outcomes by enabling timely adjustments in management. While initial studies have explored quantitative CEUS parameters to assess disease activity in Crohn’s disease, these techniques remain primarily within the research domain [22,23,30]. There are currently no universally accepted quantitative thresholds for grading inflammation severity or defining clinical cutoffs for treatment response. Nevertheless, the reproducibility and potential for objective assessment make quantitative CEUS a promising adjunct in the future of noninvasive disease monitoring. Ongoing multicenter efforts and standardization of acquisition protocols are needed before widespread clinical implementation. As research continues to refine these quantitative approaches, CEUS holds promise as a robust adjunct to conventional imaging modalities in the comprehensive management of Crohn’s disease [22,23,29,30].

Another promising area for the utilization of CEUS is in the evaluation of neonates suspected of having NEC. Although abdominal radiography has long been the imaging modality of choice in diagnosing and following NEC, several recent works have noted US’s increased sensitivity to NEC findings, including bowel wall pneumatosis and pneumoperitoneum [26,31,32]. In addition, US can also demonstrate findings not identified on abdominal radiography, such as the absence of peristalsis, wall thickness, and perfusion [33,34,35,36]. The most important outcome determinants in NEC are the presence of intestinal perforation and the extent of bowel necrosis. The presence of these critical factors often necessitates surgical intervention, which is associated with increased morbidity and mortality. Assessing bowel perfusion and viability using Doppler US can sometimes be challenging, particularly in neonates connected to oscillating ventilators, where patient motion complicates imaging. CEUS, however, maintains image quality despite motion, making it a valuable modality in the evaluation of NEC [26]. A case series by Benjamin et al. highlighted the utility of CEUS in scenarios where high-frequency oscillators limited the evaluation of color Doppler information, as the apparent vascularity on Doppler was likely confounded by rapid breathing and motion [25,26]. In such cases, CEUS provides excellent bowel perfusion evaluation with less sensitivity to motion. An additional utility of CEUS is in the assessment of bowel perfusion in the setting of intussusception. In a series of case reports of adult patients presenting with intussusception, CEUS showed a complete lack of perfusion in the invaginated intussusceptum, indicating ischemia and warranting immediate surgery [37]. However, these findings are yet to be explored in children.

### 3.3. Kidney

**Figure 4:**
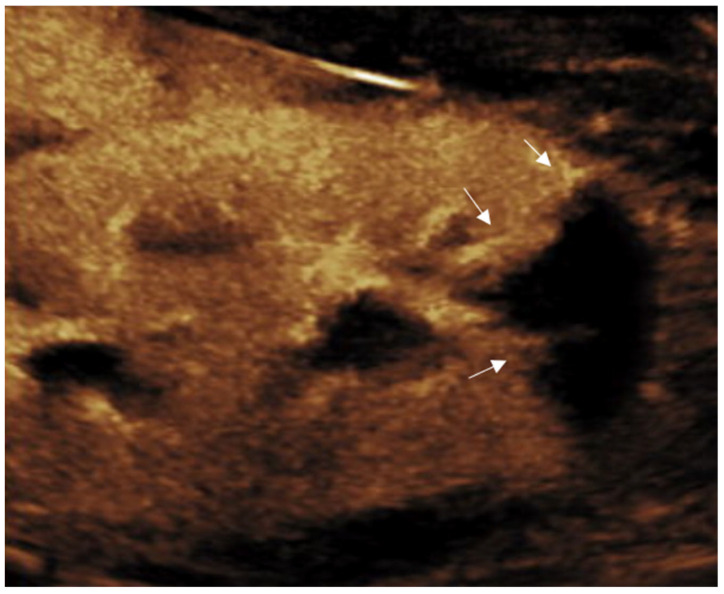
An 11-year-old girl who had recently undergone left lower renal subsegmental artery embolization. Post-procedure contrast-enhanced renal ultrasound shows a lack of parenchymal enhancement in the lowermost portion of the left kidney (arrow), suggestive of the absence of perfusion.

In the realm of pediatric kidney diseases, CEUS has proved essential in the diagnosis and characterization of various renal lesions [38,39]. The European Society of Paediatric Radiology recommends CEUS for assessing various renal pathologies in children, including VUR, complicated infections, cysts, tumors, and renal trauma [40]. One important role of CEUS pertaining to the kidney is in the assessment of renal vasculature and perfusion status. CEUS has proved relevant in evaluating focal renal stenosis and thrombosis. CEUS’s quantitative analysis capabilities, offering objective data on renal perfusion and function, are particularly beneficial in chronic kidney disease management, allowing for the monitoring of functional changes and treatment efficacy over time [38]. In this context, CEUS could potentially be used to assess microvascular perfusion patterns before and after renal angioplasty in pediatric patients [41]. CEUS has been used to evaluate changes in renal cortical blood perfusion after percutaneous transluminal renal angioplasty and stenting for severe atherosclerotic renal artery stenosis [41]. This method allows for a non-invasive assessment of renal perfusion changes following the procedure, which is critical in managing patients with severe renal artery stenosis (Figure 4). Similarly, the role of CEUS extends to the monitoring of renal transplants, a crucial aspect of post-transplant care in children. Through its ability to assess graft perfusion and detect early signs of complications like rejection or vascular stenosis, CEUS contributes significantly to the prognosis and management of renal grafts [42]. In cases of acute rejection, CEUS facilitates the evaluation of microvascular perfusion within the renal allograft, often revealing perfusion defects due to inflammatory or ischemic changes. Studies have demonstrated that CEUS can detect irregularities in renal perfusion with high sensitivity and specificity. For instance, a study reported a sensitivity of 85.7% and specificity of 100% for CEUS in diagnosing vascular rejection in renal transplant patients [43].

Similarly, CEUS is effective in identifying transplant renal stenosis by visualizing stenosed vessels and assessing downstream perfusion defects. It allows for real-time imaging of blood flow dynamics without the need for nephrotoxic contrast agents. Research indicates that CEUS can complement standard sonographic examinations in evaluating transplant renal stenosis, providing a non-invasive means to assess parenchymal kidney graft perfusion and the degree of stenosis [44].

CEUS has emerged as a valuable imaging modality for tumor characterization, particularly in assessing vascularity and distinguishing between benign and malignant lesions [45,46,47,48]. In adult populations, studies have demonstrated CEUS’s utility in evaluating renal masses, providing real-time imaging of lesion perfusion, and aiding in differentiating between various renal pathologies. In pediatric imaging, while specific enhancement patterns that reliably differentiate malignant from benign renal lesions are still under investigation, CEUS has shown promise. The European Society of Paediatric Radiology has issued recommendations supporting the use of CEUS for evaluating focal renal lesions in children, emphasizing its advantages in avoiding ionizing radiation and its superior contrast resolution [39,40]. CEUS has been shown to be useful in evaluating small focal lesions lacking enhancement. Small (<1 cm) solid lesions are often indistinguishable from cysts on CT and MRI. In these situations, CEUS might be useful because of its superb contrast resolution. Additionally, CEUS has been utilized in pediatric oncology for various applications, including the evaluation of tumor vascularity and guidance during interventional procedures. Its real-time imaging capabilities and safety profile make it a valuable tool in the pediatric population [49].

CEUS is also useful in evaluating complicated renal infections, particularly for differentiating between focal nephritis and renal abscesses [50]. On CEUS, focal nephritis typically appears as a hypoechoic, poorly perfused region during the arterial and parenchymal phases [51,52]. Importantly, some vascularity is usually preserved within the inflamed tissue, which helps differentiate it from abscesses. Renal abscesses, by contrast, are characterized by completely avascular areas on CEUS due to necrosis and purulent contents. These lesions often show surrounding hyperemia due to an inflammatory response, providing a clear distinction from focal nephritis. This differentiation is critical, as abscesses often require invasive interventions such as percutaneous drainage or surgery, whereas focal nephritis can be managed effectively with antibiotic therapy [51]. CEUS has demonstrated significant utility in diagnosing and characterizing acute pyelonephritis (APN), particularly in focal and multifocal forms. APN can appear on CEUS as wedge-shaped or rounded hypoechoic regions in the renal cortex or cortex and medulla. These areas typically exhibit diminished enhancement during the arterial and parenchymal phases, becoming more evident during the late parenchymal phase [53]. Unlike conventional US, CEUS can detect subtle perfusion abnormalities, providing a more precise visualization of inflammatory lesions.

Traditional Doppler ultrasound (US) findings in acute pyelonephritis (APN) often reveal hypovascular or avascular regions corresponding to inflammation-induced hypoperfusion [54]. However, Doppler imaging has limited sensitivity, detecting abnormalities in only 25–50% of cases [55]. Contrast-enhanced ultrasound (CEUS) overcomes these limitations by providing a detailed evaluation of the microvasculature, allowing for earlier detection of parenchymal changes and improved diagnostic accuracy. Studies have demonstrated that CEUS achieves a sensitivity of 97% and a specificity of 80% in identifying pyelonephritic lesions [56].

Future applications of CEUS in pediatric nephrology are promising, with ongoing research and technological advancements anticipated to expand its clinical utility.

### 3.4. Pancreas

Another off-label use of CEUS is the evaluation of acute and chronic pancreatic conditions, along with the characterization of pancreatic masses [57]. Benign pancreatic lesions show isoenhancement with clear borders [57,58]. Conversely, malignant lesions show a delayed enhancement with earlier washout compared to normal pancreatic parenchyma [42,59].

**Figure 5:**
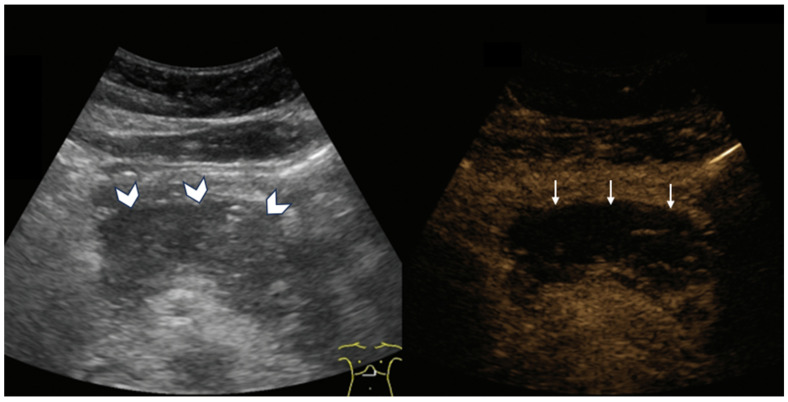
Gray scale (**left**) and contrast-enhanced (**right**) pancreatic ultrasound shows enlarged and heterogeneous pancreas (arrowhead) and non-enhancement of the almost entire pancreatic parenchyma (arrow), revealing extensive necrosis in a case of acute pancreatitis [61].

Conventional US with color Doppler is usually the primary modality for evaluating acute pancreatitis. In this modality, acute pancreatitis may present as an enlarged, edematous pancreas with peripancreatic fluid. However, identifying necrotic areas in acute necrotizing pancreatitis can be challenging using this modality alone. Studies conducted in adult populations have demonstrated that CEUS is comparable to the reference methods (CT and/or MRI) and can be considered a reliable, first-line modality for evaluating acute pancreatitis [59,60]. In acute pancreatitis, CEUS can detect pancreatic necrosis by identifying non-enhancing areas within the pancreas, indicating a lack of perfusion (Figure 5) [59,61]. Additionally, CEUS can reveal peripancreatic fluid collections and complications such as abscesses or pseudocysts, aiding in the assessment of disease severity. CEUS can serve as a valuable imaging modality, especially when CT is contraindicated, providing real-time evaluation of pancreatic perfusion and aiding in the management of acute pancreatitis. Additionally, in chronic pancreatitis, reduced blood flow can be used as a proxy for progressive fibrosis seen as disease severity increases [62].

### 3.5. Urinary Tract

VUR is a pathological condition that is relatively common in children and is characterized by uni- or bilateral reflux of urine from the urinary bladder [63]. If not properly recognized and treated, the resulting repeated urinary tract infections could lead to renal failure [63]. Currently, contrast-enhanced voiding ultrasonography (CeVUS) is indicated in children following recurrent bouts of urinary tract infections, first attack of febrile urinary tract infection in children below 1 year of age, and follow-up of a diagnosed VUR [63].

**Figure 6:**
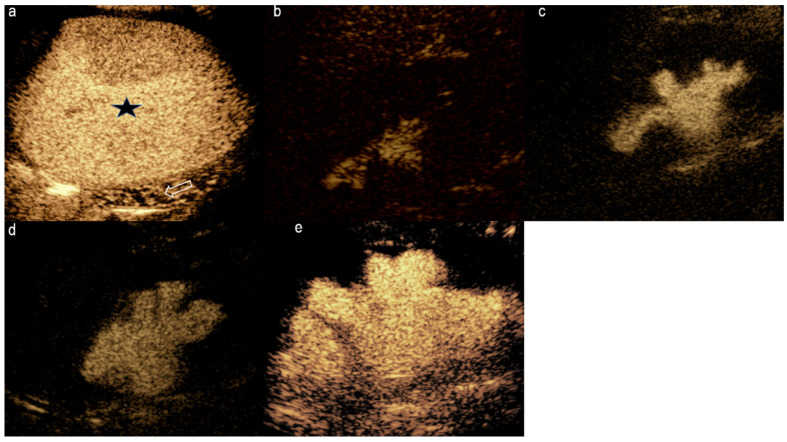
Contrast-enhanced urosonographic staging of vesicoureteric reflux. Images acquired from different children with varying degrees of reflux. (**a**) Grade I reflux: Contrast limited to the distal ureter. (**b**) Grade II reflux: Contrast extending to a non-dilated pelvicalyceal system. (**c**) Grade III reflux: Contrast seen in mildly dilated pelvicalyceal system with preserved calyceal contour. (**d**) Grade IV reflux: Contrast in moderately dilated pelvicalyceal system with blunted fornices but preserved papillary impression. (**e**) Grade V reflux: Contrast seen in severely dilated collecting system with fornices blunted and lost papillary impression. Black star: contrast filled bladder. White arrow: distal ureter.

Ntoulia and colleagues [12] have described in detail the steps involved in CeVUS in evaluating VUR, including the dosing and administration of contrast, the scan settings, and techniques. Initially, a conventional kidney and urinary tract US is performed for structural assessment. Then, after emptying the urinary bladder with a Foley catheter, a diluted contrast medium mixed with normal saline is introduced slowly. The urinary bladder and kidneys need to be constantly scanned during bladder filling and voiding so that any evidence of reflux of microbubbles into the ureter and, potentially, to the pelvi-calyceal system can be visualized to confirm the diagnosis of VUR. VUR is a dynamic process that occurs intermittently, sometimes requiring repeated and prolonged examinations [12,63]. One advantage of CeVUS is its non-utilization of ionizing radiation, allowing repeated or prolonged examination with no associated risks of exposure to ionizing radiation. A comparison between CeVUS and voiding cystourethrogram (VCUG) revealed similar performance in diagnosing and grading VUR grade II and above, with no risk of missing high-grade reflux [64]. Graded classification of VUR on CeVUS is similar to VCUG, allowing comparison between the two modalities (Figure 6) [12].

In conclusion, considering the excellent safety profile, diagnostic performance, absence of ionization radiation, and the invention of more stable UCA allowing robust assessment, it is our opinion that we may see a gradual replacement of VCUG by CeVUS in the evaluation of VUR.

### 3.6. Trauma

**Figure 7:**
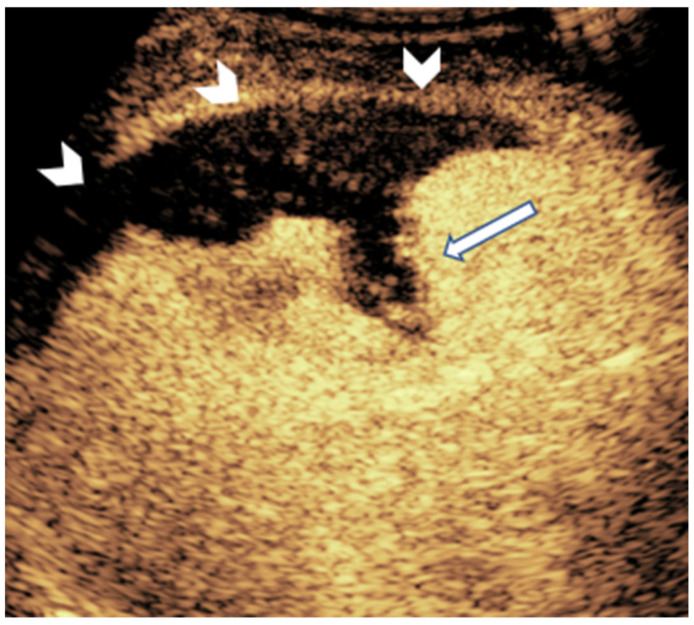
A contrast-enhanced splenic ultrasound in a 9-year-old boy with a history of abdominal trauma shows non-enhancing parenchymal (arrow) and subcapsular (arrowhead) areas representing parenchymal laceration and subcapsular hematoma, respectively.

Trauma is among the leading causes of death in children and young adults [65,66]. Focused assessment with sonography for trauma (FAST) is used to evaluate children who have sustained blunt abdominal trauma. The identification of significant hemoperitoneum in a hemodynamically unstable child warrants urgent surgical intervention [67]. However, because of FAST’s limitation in showing the specific site of organ laceration and the total extent of the sustained injury, its overall impact on the clinical management of patients has been limited [67]. CEUS overcomes this limitation by enabling the direct visualization of organ laceration and hematoma [68]. Laceration and hematoma appear as areas of non-enhancement on CEUS (Figure 7) [4,5]. A review of the diagnostic accuracy of CEUS in adults and children with blunt abdominal trauma showed a sensitivity of 98.1% and a false positive rate of 1.8% [69]. These qualities of CEUS make it a potential alternative to CT in the evaluation of hemodynamically stable children with blunt abdominal trauma.

### 3.7. Spleen

Multiple splenic conditions have been evaluated using CEUS. Normal splenic tissue will show an inhomogeneous “tiger” enhancement of the parenchyma in the arterial phase, with homogeneous persistent enhancement in the parenchymal phase [57]. Accessory spleens, which have been described to be present in 10–30% of the general population [70], can mimic enlarged hilar lymph nodes or solid tumors in the pancreas, kidney, stomach, and peritoneum [71,72,73,74]. The typical similar appearance of the accessory spleen to the major spleen in all phases of contrast allows confident identification.

**Figure 8:**
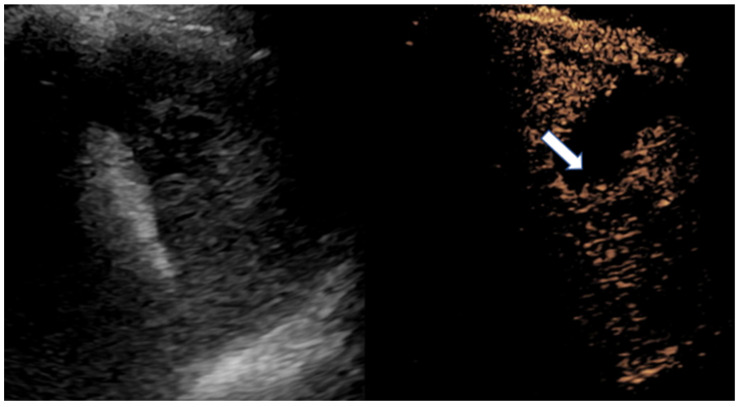
A 16-year-old-girl with acute myelogenous leukemia and multiorgan failure developed a peripheral, wedge-shaped, non-enhancing splenic lesion (arrow) on CEUS, likely denoting splenic infarct.

Splenic infarctions are most commonly present in children with prothrombotic conditions, including myeloproliferative diseases, glycogen storage diseases, bacterial endocarditis, and thromboembolic disorders [75]. The classical appearance of a splenic infarct is a peripheral wedge-shaped area within the splenic parenchyma, pointing toward the hilum (Figure 8) [76]. These lesions can be single or multiple, with the latter most commonly related to embolic conditions [57]. In CEUS, the infarct appears as a complete lack of enhancement, especially during the late phase [77]. Complications such as splenic pseudoaneurysms will appear in the arterial phase as a focal, contained accumulation of contrast [57]. Moreover, liquefaction of the infarcted tissue will appear as non-enhancing intrasplenic or perisplenic fluid collections [78]. An example of CEUS in diagnosing splenic infarction can be seen in a case report of a patient with infectious mononucleosis caused by Epstein–Barr virus, EBV [77]. In this instance, CEUS characterized the lesions as non-perfused tissue, consistent with splenic infarctions.

**Figure 9:**
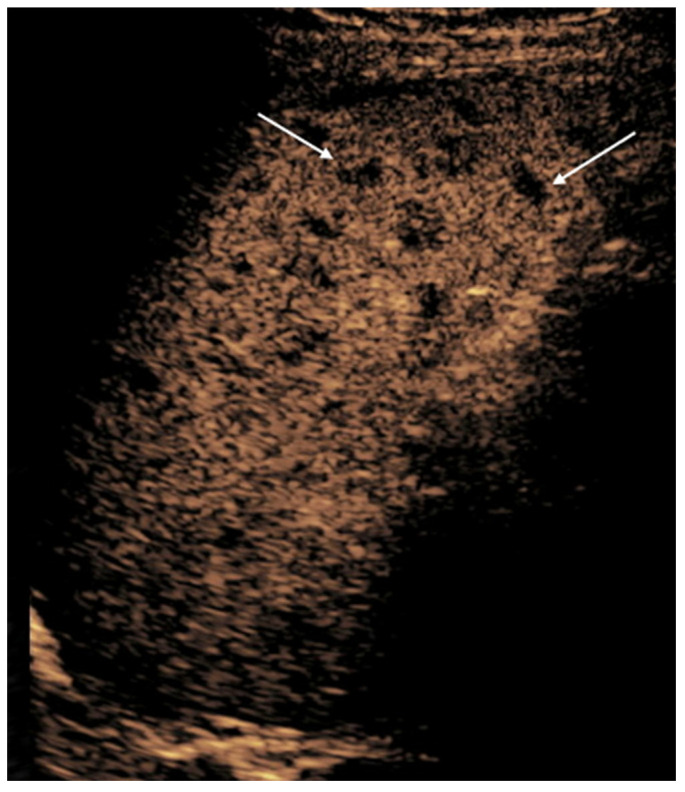
CEUS of the spleen in a 15-year-old boy with hepatosplenic candidiasis shows multiple non-enhancing nodules (arrow) suggestive of microabscesses.

Systemic infections can also produce splenic abscesses via hematogenous spread [79]. Typically, pyogenic abscesses can be detected as complex fluid collections with perilesional hyperemia seen in Doppler US. However, CEUS is particularly useful for depicting disseminated microabscesses that can be difficult to detect on conventional US (Figure 9). The lesions usually appear as a non-enhancing focus having a peripheral irregular rind of hyperenhancement after contrast injection; this perilesional enhancement can allow their differentiation from simple cysts [78].

Vascular and lymphatic malformations adjacent to the spleen, although rare, are usually detected incidentally during childhood [57]. Lymphatic malformations usually appear as multiple cystic masses of variable size that do not show internal enhancement during a CEUS examination [80]. Splenic hemangiomas are characterized by blood-filled spaces separated by fibrous septa or splenic pulp tissue. These lesions can be divided into cavernous hemangiomas (low-flow venous malformations) and capillary hemangiomas (high-flow malformations). CEUS will demonstrate hyper-, iso-, or heterogeneous hypoenhancement in the arterial phase and remain isoenhancing in the late phase [81].

While primary splenic malignancies are very rare in adults and children [82], splenic metastases are more frequent and are usually related to lymphomas, leukemia, and plasma cell malignancies [57]. In conventional US, the spleen can appear heterogeneous with the presence of various small hypoechoic nodular lesions; CEUS can distinguish these lesions as malignant by showing a variable enhancement pattern in the arterial phase and progressive or rapid washout to marked hypoenhancement in the parenchymal phase [83,84]. CEUS significantly enhances the detection of isoechoic lesions, often missed on unenhanced imaging, especially in cases of inhomogeneous splenic parenchyma or focal abnormalities. This is particularly valuable for patients presenting with left upper quadrant pain or a history of localized trauma, as CEUS can reveal pathology not evident with standard US. The technique also provides specific insights into lesion type, with benign lesions typically showing no enhancement or rapid enhancement with persistent late-phase enhancement, while malignant lesions often display arterial phase hypoenhancement followed by microbubble washout in the late phase [85].

### 3.8. Scrotum

US is widely employed for evaluating scrotal pathologies because of the organ’s superficial location, making it an ideal specimen for evaluation under a high-frequency linear transducer. Using CEUS to evaluate the scrotum is a recent but promising development. Although conventional US has already achieved acceptable accuracy in diagnosing common scrotal pathologies, CEUS can offer additional imaging information that historically would have required the child to undergo MRI [86,87].

**Figure 10:**
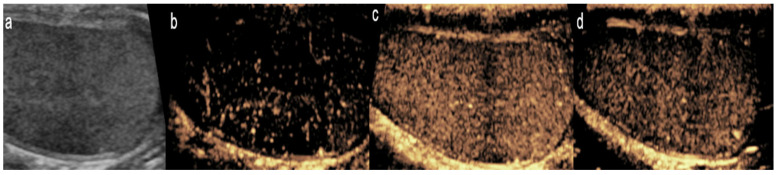
Normal grayscale and enhancement pattern of the testis in an 11-year-old boy. (**a**) Grayscale. (**b**) Arterial phase showing early linear arterial enhancement. (**c**) Parenchymal phase showing homogenous testicular enhancement. (**d**) Gradual washout.

CEUS of the scrotum is performed after a baseline conventional US examination. After administration of UCA, the arteries are the first structures to enhance [87,88]. This is followed by a rapid and diffuse filling-in of the parenchyma up to a period of 90 s [88]. Normal testicular parenchyma has a striated enhancement pattern and washout with fading of the enhancement occurs within 2–3 min (Figure 10) [86,87,88].

Testicular torsion is one of the scrotal pathologies in which the clinical utility of CEUS is tested. Testicular torsion is a common scrotal pathology in children, especially adolescents [88]. It is caused by an abrupt, complete, or partial twisting of the vascular pedicle of the testicle contained within the spermatic cord, resulting in compromised circulation [86]. Considering the short window for intervention [86] and the lack of data supporting the superior performance of CEUS compared to conventional US [88], routinely performing CEUS in all children suspected of having testicular torsion is not recommended. However, there are two situations where CEUS can be helpful. The first circumstance is to rule out torsion in young children, who typically have small testicular volume with normal minimal baseline perfusion, making conclusive exclusion of torsion using color Doppler difficult [89]. CEUS inherently has a high sensitivity for perfusion, providing improved diagnostic performance in this age group. The other is in atypical situations where recurrent episodes of torsion–detorsion cause focal segmental infarcts, which are sometimes indistinguishable from focal testicular masses [90]. Without the classic wedge-shaped outline of the segmental infarcts, infarcts can mimic hypovascular testicular masses [87]. In times of uncertainty, using CEUS can potentially be helpful by displaying segmental infarcts as avascular testicular areas separated from adjacent normal testicular lobules by enhancing vessels [86,90]. While elastography can offer additional information about tissue stiffness, it is typically performed separately from CEUS studies to avoid any theoretical risks. When performed in appropriate clinical settings and with sufficient time between modalities, elastography may enhance diagnostic confidence in differentiating infarction from neoplasm [91,92].

In the differentiation of testicular abscesses from masses, CEUS also has proven utility. A focal testicular abscess appears on CEUS as a central area of no contrast uptake surrounded by a peripheral rind of enhancement [86,89,90]. This starkly contrasts with the solid vascular appearance of most testicular tumors [87]. However, the distinction between benign and malignant masses in children using CEUS is not yet determined, warranting further study.

Finally, the role of CEUS in the evaluation of scrotal trauma has also been well documented. It is beneficial in severe scrotal injuries resulting in testicular fracture or rupture. Although visualized in only 17% of cases on conventional US, testicular fractures appear as a linear hypoechoic avascular cleft traversing the testicular parenchyma [86]. When this parenchymal cleft extends to the outer testicular lining and disrupts the tunica albuginea, it results in testicular rupture. A delay in or absence of surgical intervention of an extensive testicular injury is associated with a higher rate of orchidectomy [86]. CEUS, when performed early within the window for therapeutic intervention, provides excellent sensitivity in assessing the integrity of the tunica albuginea and fracture lines, facilitating early surgical intervention [87]. CEUS has also been helpful in surgical management by providing clear planes between salvageable and non-salvageable avascular parts of the testis [86,93,94].

## 4. Ultrasound Elastography (UE)

Palpation is one of the cardinal elements of physical examination, allowing the clinician to evaluate and characterize a multitude of superficial masses; however, it is suboptimal for lesions located at depth. UE allows the assessment of the consistency of a focal lesion or an organ in general. Central to the concept of elastography is the physical property of tissue deformation following pressure application. Rigid structures exhibit less lengthening under pressure and, therefore, less elasticity. Correlational analysis of the elasticity of different tissues makes quantitative estimation of normal ranges for different organs possible. The degree of tissue deformability can be represented in a color code or numerical value, making deviations from established standards identifiable [92,95].

UE can broadly be classified into strain elastography and shear-wave elastography (SWE). In strain elastography, an external manual mechanical compression is applied to induce tissue deformation, whereas in SWE, the US machine produces the strains in the form of an acoustic radiation pulse [88]. The quantitative results acquired are vendor-system specific, so comparison cannot be made across machines.

## 5. Clinical Applications of Elastography

### 5.1. Liver

**Figure 11:**
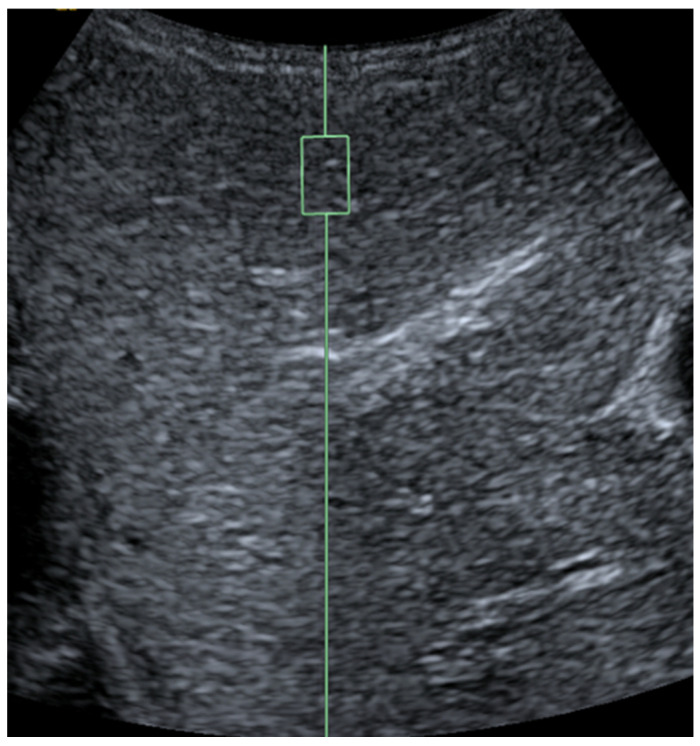
Congenital hepatic fibrosis in a 5-year-old girl. Mean shear wave elastography at the right lobe of the liver measured 3.9 m/s. The normal pediatric liver stiffness value using this ultrasound scanner and technique is approximately 1.16 m/s (SD +/− 0.14 m/s). Therefore, the finding is suggestive of fibrosis.

UE, particularly SWE, offers a robust, non-invasive approach to assessing liver stiffness—a critical marker for liver diseases like fibrosis and inflammation (Figure 11) [96]. SWE quantifies liver stiffness in kilopascals (kPa), where higher values typically indicate increased fibrosis or stiffness. In children, liver stiffness values below 6.0–6.5 kPa are generally considered normal, with values above 8.0–10.0 kPa raising concerns for significant fibrosis or cirrhosis, depending on the clinical context [97,98]. This quantitative precision is especially valuable amid the rising prevalence of obesity-related non-alcoholic fatty liver disease (NAFLD) in children, where elevated stiffness values may indicate steatosis (fat accumulation) and inflammation severity [97,98]. By enabling early and accurate detection, UE plays an instrumental role in pediatric liver disease management, reducing the reliance on invasive biopsies.

Pediatric liver disease has unique etiologies compared to that in adults, with conditions such as cystic fibrosis-associated liver disease, various metabolic liver disorders, Gaucher’s disease, Wilson’s disease, a1-antitrypsin deficiency, and glycogen storage disease presenting distinct fibrosis risks [99]. UE emerges as a simpler, less invasive option for monitoring these chronic liver diseases, often eliminating the need for repeated biopsies, thereby optimizing patient care and follow-up [100].

In clinical applications, UE has demonstrated a high ability to detect cirrhosis and differentiate between healthy and fibrotic liver tissues, with normal stiffness values remaining consistent across studies in children without liver disease [101]. Both transient elastography and 2D-SWE show strong correlations with histological assessments of fibrosis. However, transient elastography’s precision is occasionally questioned, particularly at higher stiffness ranges [102,103]. Notably, liver inflammation or elevated transaminase levels may confound fibrosis staging via UE by transiently increasing stiffness values. Despite this, UE remains a promising tool for diagnosing advanced fibrosis in children with viral hepatitis, showing good alignment with histological findings in hepatitis B and C cases [104,105,106,107,108].

For cystic fibrosis-related liver disease, UE proves reliable for fibrosis assessment, with stiffness values above 7.0–8.0 kPa often indicating progressive fibrosis [109]. UE is recommended for regular follow-up in these patients [109]. Similarly, in children with biliary atresia after the Kasai procedure, UE has demonstrated high diagnostic accuracy. Combined with serologic markers or used to inform biopsy site selection, UE enhances fibrosis monitoring in this group [110,111,112]. Beyond fibrosis, UE holds clinical relevance across various pediatric liver conditions, such as NAFLD [113], short bowel syndrome [114], portal hypertension [115], post-transplant graft fibrosis [116,117], autosomal recessive polycystic kidney disease [118], and Wilson’s disease, where reductions in stiffness values reflect treatment efficacy [119,120].

UE has also been utilized to evaluate liver stiffness as a marker of potential hepatotoxicity, demonstrating its effectiveness in monitoring liver toxicity risk in patients receiving treatments such as methotrexate [121].

While UE is highly beneficial, it is not without limitations. The technique’s accuracy can be influenced by factors such as the child’s body size, the presence of ascites, and bowel gas. Furthermore, variations in machine settings and operator experience can lead to inconsistent results. The lack of standardized reference values specifically for the pediatric population also poses a challenge in interpreting the results [122]. Consistent with adult data, liver stiffness measurements tend to be lower in the right liver lobe and show minimal sensitivity to body mass index. While intra-operator reproducibility in SWE is generally high, younger children—especially those under five—may require more acquisitions for reliable results. Furthermore, inflammation can confound fibrosis assessment, and SWE cannot reliably distinguish between adjacent fibrosis stages. Variability in liver stiffness measurements across liver disease etiologies and US systems limits the generalizability of results, and there remains insufficient comparative data between SWE and serology to fully assess the degree of inflammation, necrosis, or deposits (e.g., iron, copper) in pediatric liver disease.

### 5.2. Renal

In conditions such as hydronephrosis, pyelonephritis, and renal scarring, elastography can offer vital information about renal parenchyma [123]. In hydronephrosis, for example, elastography can help distinguish between obstructive nephropathy and a dilated but non-obstructed collecting system—a crucial factor in deciding whether intervention is required, and one that was previously best assessed using functional nuclear medicine studies, which required concomitant radiation exposure [98]. Obstructive nephropathy typically results in increased renal parenchymal stiffness due to interstitial fibrosis, inflammation, and elevated intrarenal pressure. In contrast, dilated but non-obstructed collecting systems generally maintain normal or near-normal tissue elasticity, as these cases lack the pathological remodeling associated with obstruction [124]. Similarly, in chronic pyelonephritis, affected areas may exhibit increased stiffness, which can be quantitatively measured through elastography, providing a more nuanced understanding of the disease extent and potentially aiding in monitoring the response to therapy [125].

Furthermore, elastography has an emerging role in chronic kidney disease in children. In chronic kidney disease, progressive fibrosis and changes in tissue composition can alter the renal parenchyma’s stiffness, which is detectable through elastographic techniques [126]. Similarly, in transplanted kidneys, elastography might play a role in detecting early signs of transplant rejection or complications, thereby improving graft survival rates and outcomes [127].

### 5.3. Pancreas

Pancreatic elastography has been utilized in some pancreatic conditions. Earlier studies evaluated the point SWE (pSWE) values in the pancreases of patients with cystic fibrosis and compared them with those obtained from healthy controls [128]. pSWE velocities were significantly lower in patients with cystic fibrosis (1.01 m/s vs. 1.30 m/s; *p*-value < 0.001). Another study evaluated SWE values in children with type 1 diabetes and healthy controls in order to understand the correlation between this value and clinical outcomes [129]. Here, SWE was positively correlated with diabetes duration, frequency of severe hypoglycemia, and cholesterol levels; conversely, SWE was negatively correlated with fasting C-peptide. These findings suggest the promising utilization of UE in the evaluation and follow-up of children with pancreatic diseases.

### 5.4. Spleen

UE can also provide additional insights during the evaluation of the spleen. For instance, spleen stiffness has been described as significantly higher in children with splenomegaly and in patients with a history of variceal hemorrhage [98,130]. Sutton and colleagues demonstrated that increased spleen stiffness was the most significant predictor of clinically significant varices in children with portal hypertension [131]. Specifically, higher spleen stiffness values were associated with a greater likelihood of variceal development. Similarly, Uchida et al. assessed acoustic radiation force impulse elastography in patients with biliary atresia after portoenterostomy and found that elevated spleen stiffness correlated significantly with increased portal vein diameter, indicating more severe portal hypertension [132]. Further supporting these findings, a systematic review and meta-analysis by Hu et al. concluded that spleen stiffness measurement is a reliable non-invasive method for evaluating portal hypertension and predicting esophageal varices in chronic liver disease patients [133]. Additionally, a study by Sintusek et al. reported that spleen stiffness is a reliable predictor of esophageal varices in children with biliary atresia, highlighting the utility of combined liver and spleen stiffness measurements in identifying high-risk varices [134]. Collectively, these studies underscore the clinical value of spleen stiffness as a predictive marker for portal hypertension severity and its complications.

## 6. Comparative Utility and Complementary Roles

CEUS and UE offer distinct, yet often complementary, diagnostic advantages across various pediatric abdominal pathologies. Their integration into clinical workflows can enhance diagnostic precision, particularly when both perfusion status and tissue stiffness are clinically relevant.

In acute clinical scenarios, CEUS is often superior due to its ability to provide real-time, dynamic visualization of blood flow and microvascular perfusion. For example, in necrotizing enterocolitis (NEC), CEUS has demonstrated value in identifying bowel wall perfusion defects even in neonates on high-frequency oscillating ventilation—where Doppler ultrasound is often limited by motion artifact (Section 3.2) [25,26]. Similarly, in renal infections, CEUS differentiates focal nephritis (hypoperfused but vascularized) from renal abscesses (avascular), informing whether antibiotic therapy or percutaneous drainage is warranted (Section 3.3) [50,51,52]. In the context of testicular trauma, CEUS accurately delineates areas of infarction or rupture, guiding urgent surgical decisions (Section 3.8) [86,87].

Conversely, ultrasound elastography excels in chronic disease monitoring, where structural alterations—such as fibrosis or stiffness—play a central role. In pediatric liver disease, shear wave elastography (SWE) can detect early fibrosis in children with cystic fibrosis, biliary atresia, or non-alcoholic fatty liver disease (NAFLD) (Section 5.1) [97,98,99,100,104,106]. SWE measurements have shown a strong correlation with biopsy-based staging and can be used for longitudinal surveillance to reduce the need for invasive procedures [98,101]. In renal imaging, UE helps distinguish obstructive hydronephrosis, which presents with increased stiffness due to fibrosis, from non-obstructive dilation (Section 5.2) [123,124]. It also holds promise in evaluating chronic kidney disease and transplant rejection through stiffness alterations of the parenchyma [126,127].

Notably, there are clinical conditions where both CEUS and UE provide complementary insights. For instance, in chronic liver diseases, CEUS can assess vascular perfusion patterns, detect focal lesions, and guide biopsy (Section 3.1) [13,16], while UE quantifies fibrosis severity, providing an overall assessment of liver health. Similarly, in the scrotum, CEUS helps differentiate infarcts from neoplasms based on enhancement patterns, while elastography can further increase diagnostic confidence by characterizing lesion stiffness (Section 3.8) [86,91].

In summary, CEUS is typically preferred in acute settings requiring perfusion assessment, whereas UE provides valuable data for fibrosis staging and chronic disease follow-up. Their combined use—when clinically appropriate—offers a multiparametric approach to pediatric imaging that can improve diagnostic yield while maintaining a non-invasive, radiation-free profile.

## 7. Conclusions

In this article, we showed the expanding role of CEUS and elastography in the evaluation of abdominal pathologies in children, ranging from acute abdominal emergencies to neoplasia. New diagnostic frontiers introduced with CEUS and elastography will likely strengthen the central position that US has in the evaluation of children. Nevertheless, a gap still exists in the degree of utilization of CEUS and elastography between adults and children, indicating the need for further scientific inquisition in the latter population.

## Data Availability

This a comprehensive review article, no new data was generated.

## References

[1] Dillman J.R., Gee M.S., Ward C.G., Drum E.T., States L.J. (2021). Imaging sedation and anesthesia practice patterns in pediatric radiology departments—A survey of the Society of Chiefs of Radiology at Children’s Hospitals (SCORCH). Pediatr. Radiol..

[2] Ntoulia A., Anupindi S.A., Back S.J., Didier R.A., Hwang M., Johnson A.M., Sommer F.G., Bellah R.D., Biko D.M., Darge K. (2021). Contrast-enhanced ultrasound: A comprehensive review of safety in children. Pediatr. Radiol..

[3] Ajmal S. (2021). Contrast-Enhanced Ultrasonography: Review and Applications. Cureus.

[4] Takahashi M.S., Yamanari M.G.I., Suzuki L., Pedrosa É.F.N.C., Lopes R.I., Chammas M.C. (2021). Use of contrast-enhanced ultrasound in pediatrics. Radiol. Bras..

[5] Squires J.H., McCarville M.B. (2021). Contrast-Enhanced Ultrasound in Children: Implementation and Key Diagnostic Applications. AJR Am. J. Roentgenol..

[6] Gumus M., Oommen K.C., Squires J.H. (2022). Contrast-enhanced ultrasound of the neonatal brain. Pediatr. Radiol..

[7] Coleman J.L., Navid F., Furman W.L., McCarville M.B. (2014). Safety of ultrasound contrast agents in the pediatric oncologic population: A single-institution experience. AJR Am. J. Roentgenol..

[8] Mao M., Xia B., Chen W., Gao X., Yang J., Li S., Zhang Y., Liu H., Zhao T., Wang L. (2019). The safety and effectiveness of intravenous contrast-enhanced sonography in Chinese children—A single center and prospective study in China. Front. Pharmacol..

[9] Piskunowicz M., Kosiak W., Batko T., Piankowski A., Połczyńska K., Adamkiewicz-Drożyńska E. (2015). Safety of intravenous application of second-generation ultrasound contrast agent in children: Prospective analysis. Ultrasound Med. Biol..

[10] Riccabona M. (2012). Application of a second-generation US contrast agent in infants and children—A European questionnaire-based survey. Pediatr. Radiol..

[11] U.S Food and Drug Administration (2021). LUMASON (Sulfur Hexafluoride Lipid-Type Amicrospheres) for Injectable Suspension, for Intravenous Use or Intravesical Use. https://www.accessdata.fda.gov/drugsatfda_docs/label/2021/203684s009lbl.pdf.

[12] Ntoulia A., Aguirre Pascual E., Back S.J., Bellah R.D., Beltrán Salazar V.P., Chan P.K.J., Damasio M.B., Darge K., Duran C., Fanti S. (2021). Contrast-enhanced voiding urosonography, part 1: Vesicoureteral reflux evaluation. Pediatr. Radiol..

[13] Anupindi S.A., Biko D.M., Ntoulia A., Poznick L., Morgan T.A., Darge K., Back S.J., Johnson A.M., Bellah R.D., Sommer F.G. (2017). Contrast-enhanced US assessment of focal liver lesions in children. Radiographics.

[14] Claudon M., Dietrich C.F., Choi B.I., Cosgrove D.O., Kudo M., Nolsøe C.P., Piscaglia F., Wilson S.R., Barr R.G., Chammas M.C. (2013). Guidelines and good clinical practice recommendations for contrast enhanced ultrasound (CEUS) in the liver—Update 2012: A WFUMB-EFSUMB initiative in cooperation with representatives of AFSUMB, AIUM, ASUM, FLAUS and ICUS. Ultraschall Med. Eur. J. Ultrasound.

[15] Schooler G.R., Squires J.H., Alazraki A., Chavhan G.B., Chernyak V., Davis J.T., Darge K., Hoffer F.A., Hwang M., Kandel J.J. (2020). Pediatric hepatoblastoma, hepatocellular carcinoma, and other hepatic neoplasms: Consensus imaging recommendations from American College of Radiology Pediatric Liver Reporting and Data System (LI-RADS) Working Group. Radiology.

[16] Fang C., Anupindi S.A., Back S.J., Franke D., Green T.G., Harkanyi Z., Hwang M., Johnson A.M., Ntoulia A., Sommer F.G. (2021). Contrast-enhanced ultrasound of benign and malignant liver lesions in children. Pediatr. Radiol..

[17] Thimm M.A., Rhee D., Takemoto C.M., Karnsakul W., Cuffari C., Guerrerio A.L., Arasu V.A., Majd M., Restrepo R., Bhargava R. (2018). Diagnosis of congenital and acquired focal lesions in the neck, abdomen, and pelvis with contrast-enhanced ultrasound: A pictorial essay. Eur. J. Pediatr..

[18] Moga T.V., Lupusoru R., Danila M., Ghiuchici A.M., Popescu A., Miutescu B., Iacob R., Niculescu D.A., Enache L.S., Comanescu A. (2024). Challenges in diagnosing focal liver lesions using contrast-enhanced ultrasound. Diagnostics.

[19] Ro E., Schooler G.R., Morin C.E., Khanna G., Towbin A.J. (2024). Update on the imaging evaluation of pediatric liver tumors from the ACR Pediatric LI-RADS Working Group. Abdom. Radiol..

[20] Chen M., Qiu M., Liu Y., Zhou W., Xie X., Zhou L. (2023). Utility of the pediatric liver contrast-enhanced ultrasound criteria in differentiating malignant and benign multifocal lesions. Pediatr. Radiol..

[21] van Wassenaer E.A., Benninga M.A., van Limbergen J.L., D’Haens G.R., Griffiths A.M., Koot B.G.P. (2022). Intestinal ultrasound in pediatric inflammatory bowel disease: Promising, but work in progress. Inflamm. Bowel Dis..

[22] Paratore M., Garcovich M., Ainora M.E., Riccardi L., Gasbarrini A., Zocco M.A. (2023). Dynamic contrast enhanced ultrasound in gastrointestinal diseases: A current trend or an indispensable tool?. World J. Gastroenterol..

[23] Gokli A., Dillman J.R., Humphries P.D., Ključevšek D., Mentzel H.-J., Rubesova E., Staboulidou I., Ntoulia A., Anupindi S.A., Back S.J. (2021). Contrast-enhanced ultrasound of the pediatric bowel. Pediatr. Radiol..

[24] Gokli A., Acord M.R., Hwang M., Medellin-Kowalewski A., Rubesova E., Anupindi S.A. (2020). Contrast-enhanced US in Pediatric Patients: Overview of Bowel Applications. Radiographics.

[25] Benjamin J.L., Dennis R., White S., Munson D., Anupindi S.A., Piskunowicz M., Ntoulia A., Back S.J., Darge K., Rubesova E. (2020). Improved diagnostic sensitivity of bowel disease of prematurity on contrast-enhanced ultrasound. J. Ultrasound Med..

[26] Hwang M., Tierradentro-García L.O., Dennis R.A., Anupindi S.A. (2022). The role of ultrasound in necrotizing enterocolitis. Pediatr. Radiol..

[27] Benchimol E.I., Fortinsky K.J., Gozdyra P., Van den Heuvel M., Van Limbergen J., Griffiths A.M. (2011). Epidemiology of pediatric inflammatory bowel disease: A systematic review of international trends. Inflamm. Bowel Dis..

[28] Chang S., Malter L., Hudesman D. (2015). Disease monitoring in inflammatory bowel disease. World J. Gastroenterol..

[29] Kucharzik T., Kannengiesser K., Petersen F. (2017). The use of ultrasound in inflammatory bowel disease. Ann. Gastroenterol..

[30] Medellin–Kowalewski A., Wilkens R., Wilson A., Ruan J., Wilson S.R. (2016). Quantitative Contrast–Enhanced Ultrasound Parameters in Crohn Disease: Their Role in Disease Activity Determination With Ultrasound. AJR Am. J. Roentgenol..

[31] Tracy S.A., Lazow S.P., Castro-Aragon I.M., Fujii A.M., Estroff J.A., Parad R.B., Feldman H.A., Lee T.C., Ngo P.D., Weldon C.B. (2020). Is abdominal sonography a useful adjunct to abdominal radiography in evaluating neonates with suspected necrotizing enterocolitis?. J. Am. Coll. Surg..

[32] Chan B., Gordon S., Yang M., Weekes J., Dance L. (2021). Abdominal ultrasound assists the diagnosis and management of necrotizing enterocolitis. Adv. Neonatal Care.

[33] Esposito F., Mamone R., Di Serafino M., Mercogliano C., Vitale V., Vallone G., Sangiovanni A., Muto M., Russo A., Grassi R. (2017). Diagnostic imaging features of necrotizing enterocolitis: A narrative review. Quant. Imaging Med. Surg..

[34] Cuna A.C., Reddy N., Robinson A.L., Chan S.S. (2018). Bowel ultrasound for predicting surgical management of necrotizing enterocolitis: A systematic review and meta-analysis. Pediatr. Radiol..

[35] D’Angelo G., Impellizzeri P., Marseglia L., Montalto A.S., Russo T., Salamone I., Romeo C., Centorrino A., Cannavò L., Gitto E. (2018). Current status of laboratory and imaging diagnosis of neonatal necrotizing enterocolitis. Ital. J. Pediatr..

[36] Mishra V., Cuna A., Singh R., Schwartz D.M., Chan S., Maheshwari A. (2022). Imaging for diagnosis and assessment of necrotizing enterocolitis. Newborn.

[37] Rafailidis V., Phillips C., Yusuf G., Sidhu P. (2017). A case of adult intussusception with greyscale, contrast-enhanced ultrasound and computerised tomography correlation. Ultrasound.

[38] Zhang W., Yi H., Cai B., He Y., Huang S., Zhang Y. (2022). Feasibility of contrast-enhanced ultrasonography (CEUS) in evaluating renal microvascular perfusion in pediatric patients. BMC Med. Imaging.

[39] Damasio M.B., Ording Müller L.-S., Augdal T.A., Avni F.E., Basso L., Bruno C., Darge K., Ključevšek D., Ntoulia A., Riccabona M. (2020). European Society of Paediatric Radiology abdominal imaging task force: Recommendations for contrast-enhanced ultrasound and diffusion-weighted imaging in focal renal lesions in children. Pediatr. Radiol..

[40] Riccabona M., Avni F.E., Damasio M.B., Ording-Müller L.-S., Blickman J.G., Darge K., Fotter R., Lee E.Y., Ključevšek D., Mentzel H.-J. (2012). ESPR Uroradiology Task Force and ESUR Paediatric Working Group—Imaging recommendations in paediatric uroradiology, part V: Childhood cystic kidney disease, childhood renal transplantation and contrast-enhanced ultrasonography in children. Pediatr. Radiol..

[41] Ran X., Lin L., Yang M., Niu G., Chen L., Shao Y., Zou Y., Wang B. (2020). Contrast-Enhanced Ultrasound Evaluation of Renal Blood Perfusion Changes After Percutaneous Transluminal Renal Angioplasty and Stenting for Severe Atherosclerotic Renal Artery Stenosis. Ultrasound Med. Biol..

[42] Franke D., Daugherty R.J., Ključevšek D., Ntoulia A., Rafailidis V., Takahashi M.S., Damasio M.B., Anupindi S.A., Back S.J., Darge K. (2021). Contrast-enhanced ultrasound of transplant organs—Liver and kidney—In children. Pediatr. Radiol..

[43] Mueller-Peltzer K., Negrão de Figueiredo G., Fischereder M., Habicht A., Rübenthaler J., Clevert D.A. (2018). Vascular rejection in renal transplant: Diagnostic value of contrast-enhanced ultrasound (CEUS) compared to biopsy. Clin. Hemorheol. Microcirc..

[44] Pan F.-S., Liu M., Luo J., Tian W.-S., Liang J.-Y., Xu M., Zhang Y., Wang H., Chen L., Zhao X. (2017). Transplant renal artery stenosis: Evaluation with contrast-enhanced ultrasound. Eur. J. Radiol..

[45] Tufano A., Drudi F.M., Angelini F., Polito E., Martino M., Granata A., Pugliese R., Ricci F., Manenti G., Bonomo L. (2022). Contrast-enhanced ultrasound (CEUS) in the evaluation of renal masses with histopathological validation—Results from a prospective single-center study. Diagnostics.

[46] Zhu J., Li N., Zhao P., Wang Y., Song Q., Song L., Zhang Y., Liu X., Chen H., Wang J. (2023). Contrast-enhanced ultrasound (CEUS) of benign and malignant renal tumors: Distinguishing CEUS features differ with tumor size. Cancer Med..

[47] Sun D., Wei C., Li Y., Lu Q., Zhang W., Hu B. (2016). Contrast-Enhanced Ultrasonography with Quantitative Analysis allows Differentiation of Renal Tumor Histotypes. Sci. Rep..

[48] Li J., Huang X., Wang L., Wang X., Li Y., Liu X., Zhang H., Chen Y., Zhou Q., Xu T. (2024). Role of contrast-enhanced ultrasound with the enhancement pattern and qualitative analysis for differentiating hypovascular solid renal lesions. Ultrasound Med. Biol..

[49] Marschner C.A., Rübenthaler J., Froelich M.F., Schwarze V., Clevert D.-A. (2021). Benefits of contrast-enhanced ultrasonography for interventional procedures. Ultrasonography.

[50] Pšeničny E., Glušič M., Pokorn M., Ključevšek D. (2022). Contrast-enhanced ultrasound in detection and follow-up of focal renal infections in children. Br. J. Radiol..

[51] Jung H.J., Choi M.H., Pai K.S., Kim H.G. (2020). Diagnostic performance of contrast-enhanced ultrasound for acute pyelonephritis in children. Sci. Rep..

[52] Rinaldo C., Grimaldi D., Di Serafino M., Iacobellis F., Verde F., Caruso M., Capasso R., Esposito M., Romeo V., Imbriaco M. (2023). An update on pyelonephritis: Role of contrast-enhanced ultrasound (CEUS). J. Ultrasound.

[53] Kim B., Lim H.K., Choi M.H., Woo J.Y., Ryu J., Kim S., Park C.M., Lee W.J., Han M.C., Song I.C. (2001). Detection of parenchymal abnormalities in acute pyelonephritis by pulse inversion harmonic imaging with or without microbubble ultrasonographic contrast agent: Correlation with computed tomography. J. Ultrasound Med..

[54] Boccatonda A., Stupia R., Serra C. (2024). Ultrasound, contrast-enhanced ultrasound and pyelonephritis: A narrative review. World J. Nephrol..

[55] Basiratnia M., Noohi A.H., Lotfi M., Alavi M.S. (2006). Power Doppler sonographic evaluation of acute childhood pyelonephritis. Pediatr. Nephrol..

[56] Nikolaidis P., Dogra V.S., Goldfarb S., Gore J.L., Harvin H.J., Heilbrun M.E., Kawashima A., Oto A., Remer E.M., Expert Panel on Urologic Imaging (2018). ACR appropriateness criteria^®^ acute pyelonephritis. J. Am. Coll. Radiol..

[57] Franke D., Anupindi S.A., Barnewolt C.E., Green T.G., Greer M.-L.C., Harkanyi Z., Johnson A.M., Ntoulia A., Back S.J., Darge K. (2021). Contrast-enhanced ultrasound of the spleen, pancreas and gallbladder in children. Pediatr. Radiol..

[58] Bartolotta T.V., Randazzo A., Bruno E., Alongi P., Taibbi A. (2021). Focal Pancreatic Lesions: Role of Contrast-Enhanced Ultrasonography. Diagnostics.

[59] Ripollés T., Martínez M.J., López E., Castelló I., Delgado F. (2010). Contrast-enhanced ultrasound in the staging of acute pancreatitis. Eur. Radiol..

[60] Ardelean M., Şirli R., Sporea I., Bota S., Martie A., Popescu A., Dănilă M., Tudor A., Lupuşoru R., Lazăr D. (2014). Contrast-enhanced ultrasound in the pathology of the pancreas—A monocentric experience. Med. Ultrason..

[61] Șirli R., Popescu A., Seicean A. (2017). Contrast-enhanced ultrasound for the assessment of pancreatic lesions. Challenges in Pancreatic Pathology.

[62] Azemoto N., Kumagi T., Yokota T., Hirooka M., Kuroda T., Koizumi M., Hiraoka A., Tokumoto Y., Tada F., Abe M. (2015). Utility of contrast-enhanced transabdominal ultrasonography to diagnose early chronic pancreatitis. Biomed. Res. Int..

[63] Sofia C., Solazzo A., Cattafi A., Chimenz R., Cicero G., Marino M.A., Bartolotta T.V., Ascenti G., D’Angelo P., Blandino A. (2021). Contrast-enhanced voiding urosonography in the assessment of vesical-ureteral reflux: The time has come. Radiol. Med..

[64] Kim D., Choi Y.H., Choi G., Lee S., Lee S., Cho Y.J., Kim J.H., Park S.H., Jang W., Seo J.K. (2021). Contrast-enhanced voiding urosonography for the diagnosis of vesicoureteral reflux and intrarenal reflux: A comparison of diagnostic performance with fluoroscopic voiding cystourethrography. Ultrasonography.

[65] Centers for Disease Control and Prevention (2021). Explore Leading Causes of Death. WISQARS Leading Causes of Death Visualization Tool. https://wisqars.cdc.gov/lcd/?o=LCD&y1=2021&y2=2021&ct=10&cc=ALL&g=00&s=0&r=0&ry=0&e=0&ar=lcd1age&at=groups&ag=lcd1age&a1=0&a2=199.

[66] Centers for Disease Control and Prevention (2021). Underlying Cause of Death, 2018–2021, Single Race Results Form. https://wonder.cdc.gov/controller/datarequest/D158;jsessionid=302F1E538CFED249AED2C78B127B.

[67] Richards J.R., McGahan J.P. (2017). Focused assessment with sonography in Trauma (FasT) in 2017: What Radiologist can learn. Radiology.

[68] Paltiel H.J., Barth R.A., Bruno C., Chen A.E., Deganello A., Harkanyi Z., Johnson A.M., Ntoulia A., Back S.J., Darge K. (2021). Contrast-enhanced ultrasound of blunt abdominal trauma in children. Pediatr. Radiol..

[69] Zhang Z., Hong Y., Liu N., Chen Y. (2017). Diagnostic accuracy of contrast enhanced ultrasound in patients with blunt abdominal trauma presenting to the emergency department: A systematic review and meta-analysis. Sci. Rep..

[70] Mortelé K.J., Mortelé B., Silverman S.G. (2004). CT features of the accessory spleen. AJR Am. J. Roentgenol..

[71] Trujillo S.G., Saleh S., Burkholder R., Shibli F., Shah B. (2022). Accessory spleen: A rare and incidental finding in the stomach wall. Cureus.

[72] George M., Evans T., Lambrianides A.L. (2012). Accessory spleen in pancreatic tail. J. Surg. Case Rep..

[73] Zugail A.S., Ahallal Y., Comperat E.-M., Guillonneau B. (2019). Splenorenal fusion mimicking renal cancer: One case report and literature review. Urol. Ann..

[74] Xu S.-Y., Sun K., Xie H.-Y., Zhou L., Zheng S.-S., Wang W. (2017). Accessory spleen located in the right parietal peritoneum: The first case report. Medicine.

[75] Wand O., Tayer-Shifman O.E., Khoury S., Hershko A.Y. (2018). A practical approach to infarction of the spleen as a rare manifestation of multiple common diseases. Ann. Med..

[76] Catalano O., Sandomenico F., Vallone P., D’Errico A.G., Siani A. (2006). Contrast-enhanced sonography of the spleen. Semin Ultrasound CT MR.

[77] Reichlin M., Bosbach S.J., Minotti B. (2022). Splenic Infarction Diagnosed by Contrast-enhanced Ultrasound in Infectious Mononucleosis—An Appropriate Diagnostic Option: A Case Report with Review of the Literature. J. Med. Ultrasound..

[78] Zavariz J.D., Konstantatou E., Deganello A., Bosanac D., Huang D.Y., Sellars M.E., Patel N., Alhasan R., Westwood M., Sidhu P.S. (2017). Common and uncommon features of focal splenic lesions on contrast-enhanced ultrasound: A pictorial review. Radiol. Bras..

[79] Radcliffe C., Tang Z., Gisriel S.D., Grant M. (2022). Splenic abscess in the new millennium: A descriptive, retrospective case series. Open Forum. Infect Dis..

[80] Roman A., Iancu C., Andreica V., Socaciu M., Anton O., Sechel R., Pop D., Mera M., Muresan M., Zaharie F. (2016). Splenic cystic lymphangioma with atypical ultrasound findings. J. Med. Ultrason..

[81] Stang A., Keles H., Hentschke S., von Seydewitz C.U., Dahlke J., Malzfeldt E., Schneider G., Bücker A., Forsting M., Huppertz A. (2009). Differentiation of benign from malignant focal splenic lesions using sulfur hexafluoride-filled microbubble contrast-enhanced pulse-inversion sonography. AJR Am. J. Roentgenol..

[82] Lei Y., Huang Q., Li X., Zheng X., Liu M. (2022). Characteristics and survival outcomes of primary splenic cancers: A SEER population-based study. Medicine.

[83] Ballestri S., Lonardo A., Romagnoli D., Losi L., Loria P. (2015). Primary lymphoma of the spleen mimicking simple benign cysts: Contrast-enhanced ultrasonography and other imaging findings. J. Med. Ultrason..

[84] Sutherland T., Temple F., Hennessy O., Lee W.-K. (2010). Contrast-enhanced ultrasound features of primary splenic lymphoma. J. Clin. Ultrasound.

[85] Omar A., Freeman S. (2016). Contrast-enhanced ultrasound of the spleen. Ultrasound.

[86] Cokkinos D.D., Partovi S., Rafailidis V., Sierrou C., Fragkouli T., Tsolaki S., Alzyoud K., Vlachou M., Kalogeropoulos I., Ntoulia A. (2023). Role and added value of contrast-enhanced ultrasound of the painful scrotum in the emergency setting. J. Ultrasound.

[87] Tenuta M., Sesti F., Bonaventura I., Mazzotta P., Pofi R., Gianfrilli D., Isidori A.M., Minnetti M., Greco E.A., Calogero A.E. (2021). Use of contrast-enhanced ultrasound in testicular diseases: A comprehensive review. Andrology.

[88] Huang D.Y., Pesapane F., Rafailidis V., Deganello A., Sellars M.E., Sidhu P.S. (2020). The role of multiparametric ultrasound in the diagnosis of paediatric scrotal pathology. Br. J. Radiol..

[89] Kitami M. (2017). Ultrasonography of pediatric urogenital emergencies: Review of classic and new techniques. Ultrasonography.

[90] Bertolotto M., Cantisani V., Valentino M., Pavlica P., Derchi L.E. (2016). Pitfalls in imaging for acute scrotal pathology. Semin. Roentgenol..

[91] Pozza C., Tenuta M., Sesti F., Bertolotto M., Huang D.Y., Sidhu P.S., Cantisani V., Greco E.A., Isidori A.M., Maggi M. (2023). Multiparametric ultrasound for diagnosing testicular lesions: Everything you need to know in daily clinical practice. Cancers.

[92] Mentzel H.-J., Glutig K., Gräger S., Krüger P.-C., Waginger M. (2022). Ultrasound elastography in children—Nice to have for scientific studies or arrived in clinical routine?. Mol. Cell Pediatr..

[93] Yusuf G., Konstantatou E., Sellars M.E., Huang D.Y., Sidhu P.S. (2015). Multiparametric Sonography of Testicular Hematomas: Features on Grayscale, Color Doppler, and Contrast-Enhanced Sonography and Strain Elastography. J. Ultrasound Med..

[94] Valentino M., Bertolotto M., Derchi L., Bertaccini A., Pavlica P., Martorana G., Serio G., Barozzi L., Belgrano E., Cova M.A. (2011). Role of contrast-enhanced ultrasound in acute scrotal diseases. Eur. Radiol..

[95] Stenzel M., Mentzel H.-J. (2014). Ultrasound elastography and contrast-enhanced ultrasound in infants, children and adolescents. Eur. J. Radiol..

[96] Dietrich C.F., Ferraioli G., Sirli R., Popescu A., Sporea I., Pienar C., Saftoiu A., Dong Y., Friedrich-Rust M., Cui X.W. (2019). General advice in ultrasound-based elastography of pediatric patients. Med. Ultrason..

[97] Habibi H.A., Cicek R.Y., Kandemirli S.G., Ure E., Ucar A.K., Aslan M., Kizilkaya M., Aydin S., Tutar O., Demir M.K. (2017). Acoustic radiation force impulse (ARFI) elastography in the evaluation of renal parenchymal stiffness in patients with ureteropelvic junction obstruction. J. Med. Ultrason..

[98] Močnik M., Marčun Varda N. (2023). Ultrasound elastography in children. Children.

[99] Forna L., Bozomitu L., Lupu V.V., Lupu A., Trandafir L.M., Adam Raileanu A., Ciobotaru-Orășanu C., Tănase A., Brănișteanu D., Pricop M. (2024). Pediatric perspectives on liver cirrhosis: Unravelling clinical patterns and therapeutic challenges. J. Clin. Med..

[100] Sönmez S., Boşat M., Yurtseven N., Yurtseven E. (2019). The role of elastography in the assessment of chronic liver disease in children. Afr. Health Sci..

[101] Andersen S.B., Ewertsen C., Carlsen J.F., Henriksen B.M., Nielsen M.B. (2016). Ultrasound Elastography Is Useful for Evaluation of Liver Fibrosis in Children-A Systematic Review. J. Pediatr. Gastroenterol. Nutr..

[102] Teufel-Schäfer U., Flechtenmacher C., Fichtner A., Hoffmann G.F., Schenk J.P., Engelmann G. (2021). Transient elastography correlated to four different histological fibrosis scores in children with liver disease. Eur. J. Pediatr..

[103] Dardanelli E.P., Orozco M.E., Lostra J., Laprida C., Lulkin S., Bosaleh A.P., Russo R., Marcolongo M., Gismondi M.I., Fassio E. (2020). Bidimensional shear-wave elastography for assessing liver fibrosis in children: A proposal of reference values that correlate with the histopathological Knodell–Ishak score. Pediatr. Radiol..

[104] Lee S., Choi Y.H., Cho Y.J., Lee S.B., Cheon J.-E., Kim W.S., Kim I.O., Yoo S.Y., Kim M.J., Lee M.J. (2021). The usefulness of noninvasive liver stiffness assessment using shear-wave elastography for predicting liver fibrosis in children. BMC Med. Imaging.

[105] Raizner A., Shillingford N., Mitchell P.D., Harney S., Raza R., Serino J., Lau E., Atkinson E., Fishman D.S., Jonas M.M. (2017). Hepatic inflammation may influence liver stiffness measurements by transient elastography in children and young adults. J. Pediatr. Gastroenterol. Nutr..

[106] Xu Z., Zhao J., Liu J., Dong Y., Wang F., Yan J., Li J., Wu Z., Zhang Y., Chen C. (2021). Assessment of liver fibrosis by transient elastography in young children with chronic hepatitis B virus infection. Hepatol. Int..

[107] Luo H., Peng S., Ouyang W., Tan Y., Jiang T., Tang L., Li X., Zhang H., Huang Y., Wang Y. (2022). Assessment of liver fibrosis by transient elastography and multi-parameters model in young children with chronic hepatitis B virus infection. BMC Infect. Dis..

[108] Galal S.M., Soror S.M., Hussien O., Moustafa E.F., Hassany S.M. (2020). Noninvasive assessment of liver fibrosis in children with chronic hepatitis C: Shear wave elastography and APRI versus liver biopsy. Arab. J. Gastroenterol..

[109] Enaud R., Frison E., Missonnier S., Fischer A., de Ledinghen V., Perez P., Reix P., Desmazes-Dufeu N., Dubus J.C., Bui S. (2022). Cystic fibrosis and noninvasive liver fibrosis assessment methods in children. Pediatr. Res..

[110] Hwang J., Yoon H.M., Kim K.M., Oh S.H., Namgoong J.-M., Kim D.Y., Kim W.S., Cheon J.E., Kim I.O., Kim J.H. (2021). Assessment of native liver fibrosis using ultrasound elastography and serological fibrosis indices in children with biliary atresia after the Kasai procedure. Acta Radiol..

[111] Caruso M., Cuocolo R., Di Dato F., Mollica C., Vallone G., Romeo V., Imbriaco M., Salvatore M., Di Paolo M., Giugliano A. (2020). Ultrasound, shear-wave elastography, and magnetic resonance imaging in native liver survivor patients with biliary atresia after Kasai portoenterostomy: Correlation with medical outcome after treatment. Acta Radiol..

[112] Zhou W., Li X., Zhang N., Liao B., Xie X., Zhang X., Chen S., Tang Y., Zhang Y., Wang W. (2021). The combination of conventional ultrasound and shear-wave elastography in evaluating the segmental heterogeneity of liver fibrosis in biliary atresia patients after Kasai portoenterostomy. Pediatr. Surg. Int..

[113] Sağlam N.Ö., Aksoy S., Kazancı S.Y., Palabıyık F., Hatipoğlu S.S., İnci E. (2021). The performance of shear wave elastography on evaluating liver changes in obese and overweight children. Turk. J. Pediatr..

[114] Lawrence A.E., Dienhart M., Cooper J.N., Lodwick D., Lopez J.J., Fung B., Danziger-Isakov L., Nadler E.P., Besner G.E., Gittes G.K. (2019). Ultrasound elastography as a non-invasive method to monitor liver disease in children with short bowel syndrome: Updated results. J. Pediatr. Surg..

[115] Kim D.W., Yoon H.M., Jung A.Y., Lee J.S., Oh S.H., Kim K.M., Kim W.S., Cheon J.E., Kim I.O., Kim J.H. (2019). Diagnostic performance of ultrasound elastography for evaluating portal hypertension in children: A systematic review and meta-analysis. J. Ultrasound Med..

[116] Vinciguerra T., Brunati A., David E., Longo F., Pinon M., Ricceri F., Smilari P., Bognanno G., Farinella E., Dalla Pozza L. (2018). Transient elastography for non-invasive evaluation of post-transplant liver graft fibrosis in children. Pediatr. Transplant..

[117] Lee C.K., Nastasio S., Mitchell P.D., Fawaz R., Elisofon S.A., Vakili K., Mazariegos G.V., Jonas M.M., Soriano H.E., Lerret S.M. (2020). Transient elastography assessment of liver allograft fibrosis in pediatric transplant recipients. Pediatr. Transplant..

[118] Hartung E.A., Wen J., Poznick L., Furth S.L., Darge K. (2019). Ultrasound elastography to quantify liver disease severity in autosomal recessive polycystic kidney disease. J. Pediatr..

[119] Hwang J., Yoon H.M., Jung A.Y., Lee J.S., Kim K.M., Oh S.H., Kim D.Y., Kim W.S., Cheon J.E., Kim I.O. (2020). Diagnostic performance of ultrasound elastography and serologic fibrosis indices for evaluation of hepatic involvement in Wilson disease. J. Ultrasound Med..

[120] Stefanescu A.C., Pop T.L., Stefanescu H., Miu N. (2016). Transient elastography of the liver in children with Wilson’s disease: Preliminary results. J. Clin. Ultrasound.

[121] Özdemir Çiçek S., Karaman Z.F., Şahin N., Paç Kısaarslan A., Poyrazoğlu M.H., Düşünsel R. (2022). Evaluation of liver elasticity with shear-wave elastography in juvenile idiopathic arthritis patients receiving methotrexate. Pediatr. Int..

[122] Tran L.C., Ley D., Bourdon G., Coopman S., Lerisson H., Tillaux C., Belarbi N., Ducou le Pointe H., Giorgi R., Franchi-Abella S. (2022). Noninvasive pediatric liver fibrosis measurement: Two-dimensional shear wave elastography compared with transient elastography. Front. Pediatr..

[123] Yoǧurtçuoǧlu B., Damar Ç. (2021). Renal elastography measurements in children with acute glomerulonephritis. Ultrasonography.

[124] Correas J.-M., Anglicheau D., Joly D., Gennisson J.-L., Tanter M., Hélénon O. (2016). Ultrasound-based imaging methods of the kidney-recent developments. Kidney Int..

[125] Maralescu F.-M., Vaduva A., Schiller A., Petrica L., Sporea I., Popescu A., Lupu A., Duta C., Sirli R., Bota S. (2023). Relationship between novel elastography techniques and renal fibrosis—Preliminary experience in patients with chronic glomerulonephritis. Biomedicines.

[126] Iyama T., Sugihara T., Takata T., Isomoto H. (2021). Renal ultrasound elastography: A review of the previous reports on chronic kidney diseases. Appl. Sci..

[127] Bolboacă S.D., Elec F.I., Elec A.D., Muntean A.M., Socaciu M.A., Iacob G., Băcilă C., Tudor A., Mitre A., Şirli R. (2020). Shear-wave elastography variability analysis and relation with kidney allograft dysfunction: A single-center study. Diagnostics.

[128] Pfahler M.H.C., Kratzer W., Leichsenring M., Graeter T., Schmidt S.A., Wendlik I., Meier C., Ott M., Mason R.A., Schmidberger J. (2018). Point shear wave elastography of the pancreas in patients with cystic fibrosis: A comparison with healthy controls. Abdom. Radiol..

[129] Salah N.Y., Madkour S.S., Soliman K.S. (2022). Pancreatic shear wave elastography in children with type 1 diabetes: Relation to diabetes duration, glycemic indices, fasting C-peptide and diabetic complications. Pediatr. Radiol..

[130] Goldschmidt I., Brauch C., Poynard T., Baumann U. (2014). Spleen stiffness measurement by transient elastography to diagnose portal hypertension in children. J. Pediatr. Gastroenterol. Nutr..

[131] Button H., Fitzpatrick E., Davenport M., Burford C., Alexander E., Dhawan A., Kelly D., McKiernan P., Hadzic N., Mirza D. (2018). Transient elastography measurements of spleen stiffness as a predictor of clinically significant varices in children. J. Pediatr. Gastroenterol. Nutr..

[132] Uchida H., Sakamoto S., Kobayashi M., Shigeta T., Matsunami M., Sasaki K., Kasahara M., Nakai T., Urushihara N., Kitagawa N. (2015). The degree of spleen stiffness measured on acoustic radiation force impulse elastography predicts the severity of portal hypertension in patients with biliary atresia after portoenterostomy. J. Pediatr. Surg..

[133] Hu X., Huang X., Hou J., Ding L., Su C., Meng F. (2021). Diagnostic accuracy of spleen stiffness to evaluate portal hypertension and esophageal varices in chronic liver disease: A systematic review and meta-analysis. Eur. Radiol..

[134] Sintusek P., Siriporn N., Punpanich D., Chongsrisawat V., Poovorawan Y. (2019). Spleen and Liver Stiffness to Detect Esophageal Varices in Children with Biliary Atresia. J. Pediatr. Gastroenterol. Nutr..

